# Effect of sodium bicarbonate ingestion during 6 weeks of HIIT on anaerobic performance of college students

**DOI:** 10.1186/s12970-019-0285-8

**Published:** 2019-04-15

**Authors:** Jieting Wang, Junqiang Qiu, Longyan Yi, Zhaoran Hou, Dan Benardot, Wei Cao

**Affiliations:** 1 College of Kinesiology, Beijing Sport University, Beijing, BJ China; 2 Department of Nutrition, Georgia State University, Atlanta, GA USA; 3 Center for the Study of Human Health, Emory University, Atlanta, GA USA

**Keywords:** Sodium bicarbonate, High-intensity interval training, Anaerobic performance

## Abstract

**Background:**

Past studies have found that sodium bicarbonate ingestion prior to exercise has a performance-enhancing effect on high-intensity exercise. The aim of this study was to investigate the effects of continuous sodium bicarbonate (NaHCO_3_) supplementation on anaerobic performance during six weeks of high-intensity interval training (HIIT).

**Methods:**

Twenty healthy college-age male participants were randomly assigned to either the HCO_3_^−^ group (SB) or the placebo group (PL), with 10 subjects in each group. Both groups completed 6 weeks (3 days/week) of HIIT with the SB ingesting an orange-flavored solution containing 15 g xylitol and 0.2 g HCO_3_^−^/kg body mass during each training day, and PL ingesting a similar beverage that was HCO_3_^−^-free. This study separated 6 weeks of training into two stages with different training intensities, with the first 3 weeks at a lower intensity than the second 3 weeks. Blood samples to measure serum HCO_3_^−^ were obtained 5 min before and 30 min after the following HIIT training sessions: Week 1, training session 1; week 3, training session 3; week 6, training session 3. Three 30s Wingate tests (WAnT) were conducted before, in the middle, and after the training and the supplementation interventions, with peak power, mean power, and fatigue index obtained during WAnT, and blood lactate and heart rate obtained after WAnT.

**Results:**

Our findings indicate the following: 1) Serum HCO_3_^−^ level of SB was significantly higher than PL (*p* < 0.05) both before and after each HIIT; 2) Relative peak power in WAnT was significantly higher in the SB group after 6 weeks (*p* < 0.01); 3) Lactate clearance rate and the lactate clearance velocity after 10 min of WAnT were both significantly higher in SB in the post-test (p < 0.01); 4) Heart rate recovery rate at 10 min after WAnT in both SB and PL after 6 weeks were significantly improved (p < 0.01 and *p* < 0.05, respectively), resulting in no difference between groups on these measures.

**Conclusions:**

These data suggest that supplementation of HCO_3_^− ^at the level of 0.2 g/kg body mass before HIIT training enhances the effect of HIIT on anaerobic performance, and improves the blood lactate clearance rate and the blood lactate clearance velocity following anaerobic exercise.

## Introduction

High-intensity interval training (HIIT) refers to a training protocol involving multiple bouts of high-intensity exercise or all-out sprints that are interspersed with recovery periods [1]. Results of previous studies support the idea that HIIT significantly promotes both aerobic and anaerobic exercise capacity [2–5]. Studies have found that aerobic capacity could be positively influenced by HIIT through an increase in oxidative enzymes [6], higher oxidative enzymes activity [7–9], better oxygen transfer to cells [10], more mitochondria per cell, and better mitochondrial function [10–12]. Tabata et al. (1996) compared the effects of endurance training and HIIT training on anaerobic abilities [2]. In this study, eight sets of 20 s high-intensity exercise, with 10 s intervals between each set, for 5 days/week were completed each training day by the HIIT group [2]. After 6-weeks of training, the anaerobic capacity of the HIIT group increased by 28%, while the endurance training experienced no significant change [2]. Although other studies assessing HIIT have used different training methods, training protocols (training equipment, training intensity and time, etc.), and subject groups, most results suggest that HIIT training effectively improves anaerobic capacity [13–16]. Studies have shown that, because of the high intensity and short duration, HIIT is characterized by an energy supply derived primarily from anaerobic metabolism, although it is known that all three energy systems support the exercise in different proportions during different exercise time periods [17–19]. The ability of maintaining the required power output is related to the capacity to continuously supply ATP by anaerobic glycolysis. The benefit of pursuing higher power output in high-intensity exercise alters the kinetics of oxygen uptake (VO_2_), which can also support anaerobic performance by diminishing the demand on relatively limited anaerobic fuel sources. The study of Tomlin et al. (2001) showed a positive relationship between aerobic fitness and power recovery from high intensity intermittent exercise [20]. It appears, therefore, that the improvement in anaerobic capacity during HIIT training is likely from the combined results of enhanced phosphocreatine energy supply capacity [21], improved glycolytic enzyme activity [22, 23], and enhanced aerobic metabolism [20].

While in a state of rest, human blood is slightly alkaline (pH ~ 7.4), while muscle is neutral (pH ~ 7.0). Constant mediation of the blood and muscle acid-base balance is one of the important requirements to assure normal cellular metabolism. By neutralizing excess acidity and/or alkalinity, buffering systems in the body attempt to maintain the pH in a desired/healthy range. A primary buffering system is the carbonic acid (H_2_CO_3_)-bicarbonate ion (HCO_3_^−^) system, which functions through the reaction below:

$$ {\mathrm{H}}^{+}+{{\mathrm{H}\mathrm{CO}}_3}^{-}\leftrightharpoons {\mathrm{H}}_2{\mathrm{CO}}_3\leftrightharpoons {\mathrm{H}}_2\mathrm{O}+{\mathrm{CO}}_2 $$

Studies have confirmed that acidosis negatively impacts the release of calcium ions during muscle contraction, the activation of electrical signal receptors, the binding of calcium ions to troponin C, and metabolic enzyme activity [24–27]. These changes have the effect of hindering ATP re-synthesis and slowing glycolysis. Acidosis can result from a drop in intracellular pH during short-duration intense anaerobic exercise. Studies have found that HCO_3_^−^ can buffer the accelerated release of hydrogen ions associated with this intense anaerobic activity, thereby lowering acidosis. In addition, the sodium ion (Na^+^) in sodium bicarbonate (HCO_3_^−^) can be beneficial by neutralizing the acid impact of the hydrogen ion (H^+^). It has also been established that ingestion of Na^+^ can increase the plasma volume [28], which could be an additional benefit to anaerobic activity by creating an enlarged buffering potential through dilution of the H^+^ concentration. Other studies also have found that acute or chronic exogenous HCO_3_^−^ may improve performance in 400 and 800 m races, 2 min sprints, the Wingate test, and other anaerobic activities [29–35].

The timing of acute HCO_3_^−^ supplementation in past studies typically ranges from 1 to 3 h prior to exercise, which significantly raises the blood HCO_3_^−^ level and pH in the blood [36]. Studies have found that the activity of H^+^ transmembrane protein increases in proportion to the rise of intracellular H^+^ concentration [37]. As a result, the H^+^ and lactate ions, which overflow from the cells due to exercise, can be buffered by HCO_3_^−^. Thus, the acid-buffering capacity of the muscles is improved while the increase of H^+^ in muscles is reduced to delay muscular fatigue.

A meta-analysis summarizing 29 studies found that supplementation of HCO_3_^−^ can improve anaerobic exercise capacity, significantly extending the time of exercise to exhaustion [38]. Studies also suggest that the greater the pH drop during exercise, the more beneficial the supplementation of HCO_3_^–^ [34, 39]. Some studies have compared the effects of acute and chronic HCO_3_^−^ supplementation on anaerobic ability, with results suggesting that chronic supplementation is more effective at increasing anaerobic exercise capacity than acute supplementation. A potential problem with HCO_3_^−^ supplementation is that an inappropriate dosage may result in acute gastrointestinal reactions, with symptoms that include nausea, stomach pain, diarrhea, and vomiting, all of which may negatively impact exercise performance [40]. Dose-response studies assessing commonly used HCO_3_^−^ doses, typically ranging from 0.1–0.5 g/kg body mass (BM), found that the most commonly used dose was 0.3 g/kg BM [38, 40–43]. Studies have also shown that chronic supplementation at doses lower than 0.3 g/kg BM results in better gastrointestinal tolerance than one-time acute and larger HCO_3_^−^ supplementation dose before exercise [44–46].

Current studies have assessed the effects of HCO_3_^−^ supplementation on HIIT exercise performance, but few studies have assessed the independent effects of HIIT and HCO_3_^−^ on anaerobic performance. There are several studies that have also assessed the impact of HIIT with HCO_3_^−^ supplementation on aerobic performance [47–49]. The studies of Jourkesh et al. (2011) [47] and Edge et al. (2004) [49] found that HCO_3_^−^ supplementation with HIIT positively affects aerobic capacity. In contrast, a study by Driller et al. (2013) found that this combination had the opposite effect, although the researchers hypothesized that the finding may have been due to the unique subject characteristics (highly trained members on national teams), whose aerobic performance was sufficiently well-developed that it would not likely be impacted through the addition of supplemental HCO_3_^−^ [48, 50, 51]. Because of this finding, we chose healthy college students as our subjects instead of highly trained athletes, to enable a better understanding of the possible impact of HCO_3_^−^ ingestion when coupled with HIIT.

The results of past studies led us to develop a research project that would assess the combination of chronic HCO_3_^−^ supplementation with HIIT training, to explore whether this intervention can effectively improve anaerobic capacity in healthy young men. We hypothesized that the combination of chronic HCO_3_^−^ supplementation during HIIT will result in an improvement of anaerobic exercise performance in this population.

## Methods

### Subjects

Healthy college-age male participants (*N* = 20; $\overline{x}$ age = 20.45 ± 0.94 yr.; $\overline{x}$ height = 1.76 ± 0.05 m; $\overline{x}$ body mass = 70.55 ± 5.65 kg; $\overline{x}$ BMI = 22.70 ± 1.60 kg/m [2], $\overline{x}$ lean mass% = 54.6 ± 2.05, $\overline{x}$ body fat% = 15.96 ± 4.03) constituted the subject pool (See Table 1). The inclusion criteria were as follows: 1) 18–24 year-old males; 2) BMI was between 18.5–23 kg/m^2^; 3) Healthy, with no hypertension, diabetes or cardiovascular risk factors, and no other diagnosed diseases; 4) Subjects had no professional training during the 6-month period prior to the onset of this experimental protocol; 5) Subjects voluntarily agreed to participate in the experiment. Approval for the study was obtained from the Institutional Review Board of Beijing Sport University (BSU IRB). Informed consent was obtained from all participants. In addition, participants were required to avoid other training protocols and supplementation during the experiment, and were asked to maintain their typical diet and activities.

**Table 1:** Subjects characteristics for two groups*

Group	Age (yr)	Height (cm)	Weight (kg)	BMI (kg/m^2^)	Lean Mass (%)	Body Fat (%)	MAP (W)
SB	20.35 ± 0.44	1.77 ± 0.05	71.52 ± 5.20	22.83 ± 1.65	46.05 ± 2.20	16.95 ± 4.21	183.55 ± 35.65
PL	20.51 ± 0.21	1.76 ± 0.05	69.57 ± 6.18	22.51 ± 1.64	45.15 ± 1.90	14.98 ± 3.80	179.67 ± 25.97

Values are means ± SD. SB=HCO_3_^−^ group, *PL=* placebo group

*There was not significant difference between two groups on these values

### Study design

The research proposal was approved by the Institutional Review Board of Beijing Sport University. All subjects were fully informed about the purposes, procedures, and potential risks of this study. Subjects signed an informed consent and completed a health history questionnaire and physical activity questionnaire at the first visit, followed by an assessment of body composition. After these steps, they performed a graded exercise testing (GXT) on a cycle ergometer to evaluate maximal aerobic power output (MAP). This MAP value was considered the subject’s basic exercise capacity for development of the individualized training plan. Subjects were randomly assigned to HCO_3_^−^ (SB) and Placebo (PL) groups (see Table 1) to enhance the possibility of group equivalence. We found no significant differences in BMI, lean mass%, body fat% and MAP between the two groups. All subjects completed 6 weeks of HIIT (3 days per week) on a cycle ergometer. The intensity of the training was set to a higher level in the second three week assessment/training period. Throughout this 6-week training period, the SB group ingested a fluid containing HCO_3_^−^ on every training day, while the placebo group ingested a similar beverage containing no HCO_3_^−^. Blood samples were taken before and after the first training, the third training in the third week, and the final training to measure serum HCO_3_^−^. A 30 s Wingate Anaerobic 30 s Cycling Test (WAnT) was conducted pre-, mid-, and post-intervention (i.e., before both HIIT and HCO_3_^−^ interventions, after three weeks of interventions, and after six weeks of interventions respectively). During the WAnT, peak power, mean power, fatigue index, blood lactate, and heart rate were recorded. Subjects were asked to keep their normal schedule on the day before all interventions, and to avoid consuming, within 2 h before the test, carbonated drinks, alcohol, caffeine or other substances that could affect the GXT, WAnT and blood test results. Subjects were also asked to avoid any strenuous exercise before GXT, WAnT, and blood test. All sports training, GXT, and WAnT were performed using a cycle ergometer. During the interventions, subjects were also asked to avoid any other professional training regimen and nutrition supplementation. Daily food consumption was recorded by subjects as a means of monitoring nutritional intake.

### Graded exercise testing

The first segment of the exercise capacity test was GXT on a cycle ergometer (starting at 40 W + 20 W·2 min^− 1^), which was the basis for all the following steps. The maximal aerobic power output was identified as the highest workload that subjects can maintain during the entire test. Below are the equations for MAP.

If subjects completed 2 min of their last stage: MAP = W_final_.

Where, MAP is the maximal aerobic power output, W_final_ is the power output (in watts) of the last complete stage that subjects performed.

If subjects did not complete 2 min of their last stage: MAP = W_final_ + (t/ 120 × 20).

Where, MAP is the maximal aerobic power output, W_final_ is the power output (in watts) of the last complete stage that subjects performed, and ‘t’ is the duration (i.e., time) that subjects performed in the uncompleted stage [52]. Polar monitors were worn by subjects to monitor heart rate. The standards for exhaustion included: 1) Heart rate reached 180 bpm/min; 2) Subjects subjectively felt tired; 3) Subjects couldn’t pedal at a constant load for more than 15 s. Subjects were considered exhausted when they simultaneously met any two of the above criteria.

### HIIT intervention

Both groups performed 6 weeks of the same HIIT training, the intensity of which was set based on MAP. This study set the first 3-week period and the second 3-week period as two distinct training stages with different training intensities, following the standard of prior research [53]. During the first 3-week period the goal was for subjects to finish 4 sets of training during training days, with a 1-min interval between each set. Every set consisted of 1 min cycling, which included 30 s cycling at an intensity of 100% MAP and 30 s at 70% MAP. The second set intensity was changed to 40 s cycling at 100% MAP, plus 20 s cycling at 70% MAP. Training was performed by subjects three times per week, with the training sessions separated by at least one rest day. Prior to the training, subjects were required to do a warm-up at 60 W for 5 min.

### HCO_3_^−^ supplementation

The HCO_3_^−^ supplementation dose for SB was 0.2 g/kg BM. HCO_3_^−^ was dissolved into 1 L drinking water with 15 g xylitol and 2 g orange flavoring to enhance the acceptability of the fluid. With the goal of lowering the potential for gastrointestinal distress, which could occur by drinking a high fluid volume too quickly, subjects were asked to consume the beverage over several opportunities during the 60–90 min before training. The beverage for PL was similar to that consumed by SB, except for containing no HCO_3_^−^.

### Wingate Anaerobic 30 s Cycling Test

Subjects performed a Wingate Anaerobic 30 s Cycling Test with a Monark 894E (Model: Ergomedic 894E, Manufacturer: Healthcare International) at pre-, mid- and post-intervention. A 1-day (24 h) interval was required between the test and the training. Subjects were instructed to take these three tests at the same time of the day. Subjects were given a 5-min standard warm-up prior to WAnT [54], including two 3–5 min all-out sprints and a constant warm-up at 1 W/kg BM. Following a 5-min warm-up, WAnT was conducted according to the standard method of Bar-Or [55]. Peak power (PP), mean power (MP) and fatigue index (PD%) of the test were calculated via Monark Anaerobic Testing software (Version: 3.3.0.0, Developed in Co-operation with HUR Labs). Heat rate was monitored by the Polar Rs800cx chest-worn heart rate monitor (Manufacturer: Polar Electro Oy) from the resting state to the recovery period for analyzing the heart rate recovery rate (HRR%). The equation for HRR%:

$$ {\mathrm{HR}\mathrm{R}}_{10}\%=\left({\mathrm{HR}}_{\mathrm{max}}-{\mathrm{HR}}_{10}\right)/\left({\mathrm{HR}}_{\mathrm{max}}-{\mathrm{HR}}_{\mathrm{rest}}\right)\times 100\% $$

Where HRR_10_% is the rate of heart rate recovery, HR_max_ is the maximum heart rate immediately after exercise, HR_10_ is the heart rate after 10 min of exercise, and HR_rest_ is the resting heart rate.

### Lactate test

Finger blood samples (10 μl) were obtained while in a non-exercise state as follows: immediately, 3 min, 5 min, 7 min, and 10 min after exercise following all three WAnT. The samples were tested via a glucose and lactate analyzer (Biosen C-Line, EKF Diagnostics), and the lactate clearance rate was predicted as follows:

$$ {\mathrm{LA}}_{10}\%=\left({\mathrm{LA}}_{\mathrm{max}}-{\mathrm{LA}}_{10}\right)/\left({\mathrm{LA}}_{\mathrm{max}}-{\mathrm{LA}}_{\mathrm{rest}}\right)\times 100\% $$

Where LA_10_% is the lactic acid clearance rate at 10 min after exercise, LA_max_ is the peak lactate value after exercise, LA_10_ is the lactic acid value at 10 min after exercise, and LA_rest_ is the value of lactic acid before exercise.

Lactate clearance velocity was predicted as follows:

$$ {\mathrm{V}}_{10}=\left({\mathrm{LA}}_{\mathrm{max}}-{\mathrm{LA}}_{10}\right)/\times \left(10-\mathrm{t}\right) $$

Where V_10_ is the lactate clearance velocity at 10 min after exercise, LA_max_ is the peak lactate value after exercise, LA_10_ is the lactic acid value at 10 min after exercise, and t is the time when peak lactate value appears.

### Serum HCO_3_^−^ test

Venous blood samples of 2 ml were collected before and 30 min after the first training, the third training in the third week, and the last training to measure the serum HCO_3_^−^ by a Mindary BS-420 automatic biochemical analyzer.

### Statistical analyses

Data were entered into Microsoft Excel 2010 and analyzed using SPSS version 22.0. Two-way repeated measures ANOVA was performed on the three tests to assess the interaction between time (before intervention, during intervention, after intervention) and the cohort (SB and PL). Simple effect analysis was performed for the horizontal comparison of between-group-factors when there was an interaction, and longitudinal comparison of within-group-factors was performed when there was no interaction. Otherwise, one-way repeated measures ANOVA and independent samples t-tests were used to respectively compare both intra- and inter-group factors, or only one of these two factors based on the main influencing effects. Statistical significance was set at *p* < 0.05.

## Results

All subjects completed 6 weeks of intervention, with no subject dropouts.

### Subjects characteristics

There were no significant differences of measured factors between SB and PL (*p* > 0.05). (see Table 1).

### HCO_3_^−^ level analysis

The alkaline reserve in SB was improved following supplementation (see Table 2). The results of all three tests showed that the serum HCO_3_^−^ of SB was significantly higher (*p* < 0.05) than that of PL both before and after training. Moreover, compared to the pre-intervention, serum HCO_3_^−^ both before and after HIIT were significantly increased (*p* < 0.01) in mid-intervention. Compared to the mid-intervention, serum HCO_3_^−^ both before and after HIIT were significantly increased (*p* < 0.05) at post-intervention in SB, while the PL showed no significant changes.

**Table 2:** Serum HCO_3_^−^ level before and after HIIT in the pre-, mid- and post-intervention

Group	Pre-intervention		Mid-intervention		Post-intervention	
	Before HIIT	After HIIT	Before HIIT	After HIIT	Before HIIT	After HIIT
SB	25.00 ± 1.06^*†*^	23.62 ± 1.07^*†*^	27.74 ± 1.04^*†* **^	25.72 ± 0.83^*†* **^	28.49 ± 0.87^†**△^	26.15 ± 0.73^†**△^
PL	21.63 ± 0.98	20.30 ± 0.58	21.31 ± 1.16	19.99 ± 0.99	21.48 ± 1.18	20.55 ± 1.24

Values are means ± SD. SB=HCO_3_^−^ group, *PL=* placebo group

^**^indicates a significant difference vs. pre-intervention (*p* < 0.05)

^△^indicates a significant difference from mid- to post-intervention (*p* < 0.05)

^†^indicates a significant difference (*p* < 0.05) vs. PL-group

### Wingate Anaerobic 30 s Cycling Test

**Fig. 1:**
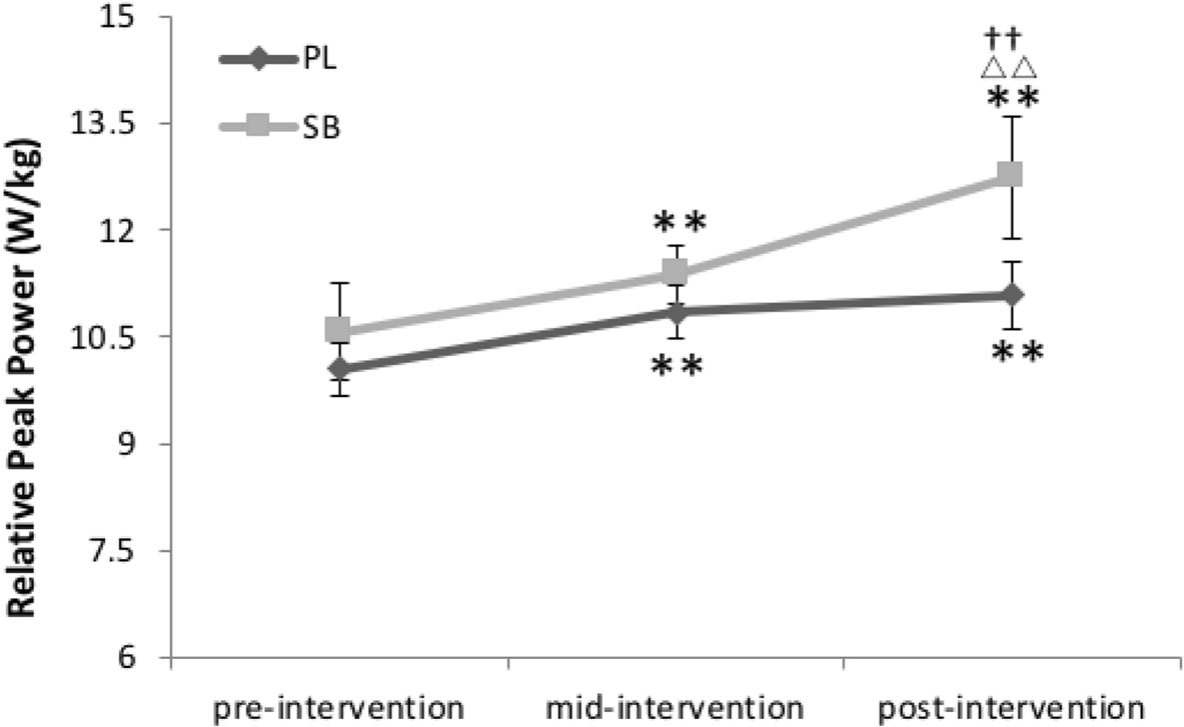
Relative peak power in the pre-, mid- and post-intervention*.* SB=HCO_3_^−^ group, PL = placebo group. ^△△^ indicates a significant difference from mid- to post-intervention (*p* < 0.01). ** indicates a significant difference from pre- to mid-interventin or from pre- to post-intervention (*p* < 0.01). †† indicates a significant difference (*p* < 0.01) vs. PL-group

The relative PP of SB was significantly higher in both the first three week period and the second three week period (*p* < 0.01). However, in PL a significant increase was only found in the first three week period (p < 0.05) (see Fig. 1). There was also a significant difference (*p* < 0.01) in PP between SB and PL after six weeks of training, with the SB having higher PP. Although the relative MP in both groups improved significantly (*p* < 0.01) after only three weeks, no significant difference was found between groups. PD% was also not different between groups. However, SB experienced a significant decrease in PD% from pre-intervention to mid-intervention (*p* < 0.05), and from mid-intervention to the post-intervention (p < 0.01). PL experienced a significant decrease of PD% after six weeks of intervention (*P* < 0.05) (see Table 3).

**Table 3:** WAnT related indexes data in the pre-, mid- and post-intervention

Variable	Group	Pre-intervention	Mid-intervention	Post-intervention
Relative peak power (W/kg)	SB	10.58 ± 0.69	11.38 ± 0.40^**^	12.78 ± 0.68^**△△††^
	PL	10.05 ± 0.38	10.85 ± 0.37^**^	11.09 ± 0.47^**^
Relative mean power (W/kg)	SB	7.53 ± 0.59	8.72 ± 0.43^**^	9.17 ± 0.40^**^
	PL	6.96 ± 0.21	7.77 ± 0.59^**^	8.25 ± 0.74^**^
Fatigue index %	SB	55.78 ± 9.24	48.95 ± 6.81^*^	44.06 ± 6.71^**^
	PL	60.57 ± 12.50	55.89 ± 12.11	53.10 ± 10.66^*^
Resting LA (mmol/L)	SB	1.92 ± 0.64	2.00 ± 1.03	1.89 ± 0.55
	PL	2.09 ± 0.65	2.17 ± 0.74	1.59 ± 0.58
LAmax (mmol/L)	SB	13.85 ± 1.40	15.71 ± 1.25	16.44 ± 1.06^**^
	PL	14.83 ± 2.49	14.69 ± 2.10	14.62 ± 2.06
LA after (10 min) WAnT (mmol/L)	SB	12.39 ± 2.14	13.43 ± 1.24	13.02 ± 1.65
	PL	13.52 ± 2.42	12.78 ± 1.72	12.45 ± 1.68
V_10_ (mmol·L^− 1^·min^− 1^)	SB	0.24 ± 0.08	0.49 ± 0.12^**^	0.62 ± 0.10^**△△††^
	PL	0.23 ± 0.07	0.39 ± 0.13^*^	0.43 ± 0.12^**^
LA_10_%	SB	11.58 ± 1.41	16.16 ± 3.01^**^	20.40 ± 1.81^**△△††^
	PL	10.59 ± 2.72	14.36 ± 2.26^**^	16.00 ± 2.07^**^
HRR_10_%	SB	65.38 ± 5.32	75.07 ± 10.20^*^	80.54 ± 4.98^**△^
	PL	63.5 ± 9.35	68.39 ± 8.77^*^	73.33 ± 8.19^*^

Values are means ± SD. *LA=* lactic acid, *LAmax=* maximal lactic acid, *WAnT=* Wingate anaerobic test, *SB=* HCO_3_^−^ group, *PL=* placebo group. (*p* < 0.05)

^△^indicates a significant difference from mid- to post-intervention (*p* < 0.05)

^△△^indicates a significant difference from mid- to post-intervention (*p* < 0.01)

^*^indicates a significant difference from pre- to mid-intervention or from pre- to post-intervention (*p* < 0.05)

^**^indicates a significant difference from pre- to mid-intervention or from pre- to post-intervention (*p* < 0.01)

^††^indicates a significant difference (*p* < 0.01) vs. PL-group

### Blood lactate

**Fig. 2:**
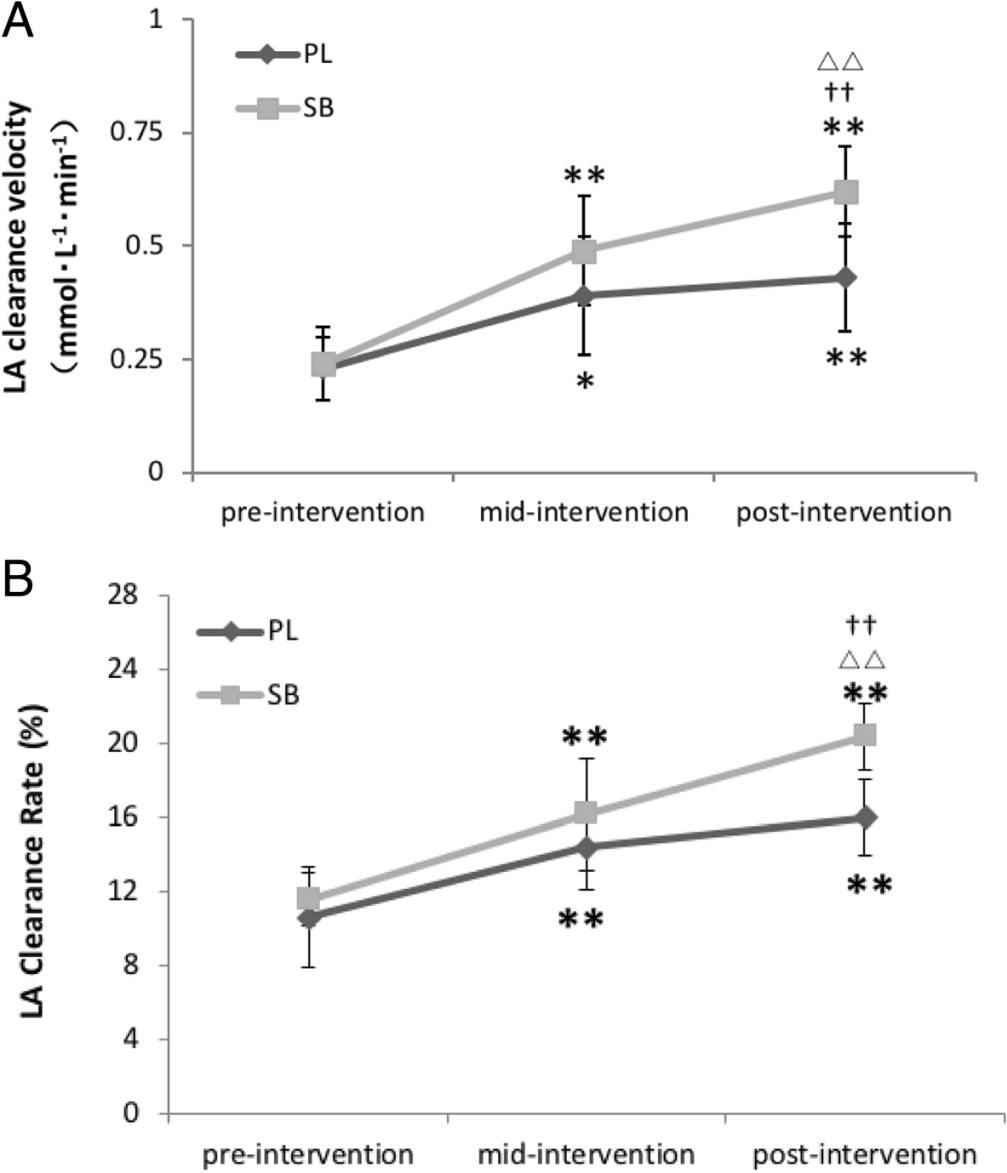
**a, b** LA clearance velocity and LA clearance rate (%) in the pre-, mid- and post-intervention. SB=HCO_3_^−^ group, *PL=* placebo group

In the three WAnT tests, there was no difference in the resting LA, either between or within groups. LA_max_ in SB increased significantly after three weeks of intervention (*p* < 0.01), while PL experienced no significant difference after six weeks. V_10_ and LA_10_% were significantly different between groups after six weeks of intervention (*p* < 0.01) (see Fig. 2).

### Heart rate

Though no significant difference of HRR_10_% was found between SB and PL during six weeks, only the SB group improved significantly from the mid-intervention to post-intervention (*p* < 0.05) (see Table 3).

## Discussion

The purpose of this study was to investigate whether HCO_3_^−^ supplementation during 6 weeks of HIIT results in a greater improvement in anaerobic capacity in healthy young men than HIIT alone. We hypothesized that chronic supplementation of HCO_3_^−^ would be in synergy with HIIT training to improve the subjects’ alkaline reserve and increase the acid-buffering capacity during exercise. We believed that this combination could postpone muscular fatigue and improve the body’s capacity to provide ATP and enhance HIIT’s effect on anaerobic performance. Our results support this hypothesis. The findings demonstrated that serum HCO_3_^−^ was significantly different between groups in all tests (*p* < 0.05). In addition, serum HCO_3_^−^ of the SB increased significantly after each stage of intervention (from pre-intervention to mid-intervention, *p* < 0.01; from mid-intervention to post-intervention, *p* < 0.05), suggesting that the alkaline reserve of subjects in SB increased after HCO_3_^−^ supplementation. These results correspond to the results of several other studies [44, 48]. According to the test results of WAnT, the significant positive changes were more evident in SB rather than in PL, which is likely due to the chronic HCO_3_^−^ supplementation intervention.

During our 6-week experiment, only one subject experienced problematic gastrointestinal effects after taking HCO_3_^−^ supplements. Through our inquiry and observation, and the fact that this was limited to a single subject, we believe that this may have been the result of a preexisting GI condition. Another possibility is that the drinking speed of the supplement was excessively fast, creating an increased gastrointestinal burden. As mentioned earlier, studies assessing HCO_3_^−^ supplementation have most commonly used HCO_3_^−^ at a concentration is 0.3 g/kg BM [38, 40–43]. It has been found that longer-term supplementation at this and lower dosage levels result in improved gastrointestinal tolerance [44–46]. The dosage applied in this study was 0.2 g/kg BM, which, according to the study by Bishop et al. (2005), could effectively elevate the blood HCO_3_^−^ level without causing negative gastrointestinal reactions [56].

According to the analyzed data, after 6 weeks of intervention relative mean power (*p* < 0.05), relative peak power (*p* < 0.05), and fatigue index (*p* < 0.01) of PL all significantly improved, implying that the effect of HIIT training alone significantly improves anaerobic capacity. This finding is consistent with earlier studies [13, 14, 57]. In the study of Naimo et al. (2015) [14], 4 weeks of HIIT training significantly increased the peak power and mean power in WAnT of college hockey players. Astorino et al.(2012) [13] conducted a short-term HIIT training for 20 males and females who often engaged in physical activity and found that both relative PP and relative MP improved significantly. However, neither of these two studies found a significant improvement in the fatigue index [13]. It is possible that this may be due to the longer training period in these two studies than in the study we designed. Therefore, by imposing more intensive physiological stimuli through the training, the transport and clearance ability of lactic acid could be more obvious, the muscle acid resistance could be stronger and, ultimately, the onset of fatigue could be slower.

Although significant differences in relative MP and PD% between groups were not observed in our study, there was a significant (*p* < 0.01) difference in relative PP over the six week intervention period. Hence, our data suggest that HCO_3_^−^ supplementation aided in the improvement of anaerobic performance. In addition, some additional changes occurred in SB, including the significant increase of relative PP in the second three week intervention period, and a significant improvement of PD% (*p* < 0.01) after six weeks. Neither of these changes was observed in PL. As a result of the mechanisms described previously, taking a sufficient dose of HCO_3_^−^ before high intensity exercise can reduce the accumulation of acid in muscle cells, delay the generation of fatigue, and enhance skeletal muscle contraction [46]. It has been demonstrated that changes in the metabolism of skeletal muscles resulting from HCO_3_^−^ supplementation before exercise improve the function of anaerobic metabolism of skeletal muscles. This alteration is beneficial for high-intensity exercise [58]. Therefore, our data suggest that, in the population studied, supplementation of HCO_3_^−^ during HIIT may have a positive impact of HIIT training on anaerobic capacity through multiple mechanisms.

The 30 s Wingate anaerobic test is heavily reliant on the ATP/CP and anaerobic glycolytic system. The peak lactate level after exercise represents the body’s maximum tolerance to lactic acid. It also reflects the capacity of the glycolytic system to produce ATP. As can be seen in Table 3, there is a significant increase in peak lactate level only in SB over six weeks of intervention (*p* < 0.01). This result suggests that the ability of glycolytic system to produce ATP is enhanced with HCO_3_^−^ supplementation. In addition, although lactic acid clearance velocity and clearance rate at 10 min after WAnT in PL improved, our findings indicate that the increase in SB is more evident on these values, resulting in a significant difference between SB and PL (p < 0.01). Similar results have been demonstrated in earlier studies [34]. This suggests that the supplementation of HCO_3_^−^ positively effects the clearance of lactate after exercise. The improvement in lactic acid clearance suggests that exogenous HCO_3_^−^ supplementation can increase the intracellular alkali reserve, slow the pH reduction in muscles, and delay the onset of fatigue.

We initially speculated that HCO_3_^−^ supplementation may promote lactic acid clearance after anaerobic exercise, reduce lactic acid accumulation and increase blood pH, which could increase the partial pressure of oxygen (PO_2_) in blood and accelerate heart rate recovery. The possible mechanism for this is that blood pH and partial pressure of carbon dioxide (PCO_2_) are pertinent to the affinity of Hb with O_2_ and the PO_2_ in the blood [59]. Both elevated pH and decreased PCO_2_ can increase the affinity of Hb with O_2_ and increase blood PO_2_ [59]. It is known that heart rate recovery and PO_2_ are positively correlated, while hypoxia and cardiac autonomic nervous dysfunction are closely related [60]. We found, however, that the rate of heart rate recovery in both groups increased after six weeks of intervention, and there was no significant difference between groups in heart rate recovery (*p* > 0.05). This is consistent with the fact that we have not found any report demonstrating an improvement of the heart rate recovery after exercise following ingestion of HCO_3_^−^. Our data suggest that oral supplementation of HCO_3_^−^ in a low dosage may positively affect heart rate recovery after exercise. While this may be the result of improved neurological regulation, there is no obvious cause of this finding.

The present study has several limitations, including no data on how to enhance the taste of supplements without affecting its efficacy. In addition, our study used a relatively small sample of only healthy young men, which limits the generalizability to women and other populations. Therefore, we would encourage a duplication of this study’s protocol with other populations in future research.

## Conclusions

Our data suggest that, in healthy young men, the combination of HCO_3_^−^ supplementation and HIIT can enhance the effect of HIIT on anaerobic performance, including improving power output, delaying fatigue onset, and improving the blood lactate clearance rate and velocity after the anaerobic exercise.

## Abbreviations

BM: Body mass
GXT: Graded exercise testing
H^+^: Hydrogen ion
H_2_CO_3_: Carbonic acid
HCO_3_^−^: Bicarbonate ion
NaHCO_3_: Sodium bicarbonate
HIIT: High-intensity interval training
HRR%: Heart rate recovery rate
MAP: Maximal aerobic power
MP: Mean power
Na^+^: Sodium ion
PCO_2_: Partial pressure of carbon dioxide
PD%: Fatigue index
PO_2_: Partial pressure of oxygen
PP: Peak power
WAnT: Wingate Anaerobic 30 s Cycling Test
